# Utilization of Internet Resources by Surgeons for Continuous Professional Development in the Era of Prevailing COVID-19 Pandemic: Trends and Obstacles

**DOI:** 10.3389/fsurg.2022.899803

**Published:** 2022-06-14

**Authors:** Sanem Guler Cimen, Asir Eraslan, Fahrettin Samil Uysal, Ahmet Emin Dogan, Alihan Kokurcan, Muhammet Sahin Yilmaz, Burhan Baylan, Sertac Cimen

**Affiliations:** ^1^ Department of General Surgery, Health Sciences University Diskapi Training and Research Hospital, Saglik Bilimleri Universitesi, Ankara, Turkey; ^2^ Department of Urology, Health Sciences University Diskapi Training and Research Hospital, Ankara, Turkey

**Keywords:** internet resources, PubMed, google scholar, surgical education, continuous professional development, digital competence

## Abstract

**Background:**

To investigate the use of internet resources by surgeons for continuing professional development (CPD).

**Results:**

This cross-sectional study was carried out between July 1, 2021, to October 31, 2021, at the Department of Medicine, Health Sciences University Diskapi Yildirim Beyazit Training and Research Hospital, Ankara, Turkey, with participants from nine surgical specialties: General surgery, neurosurgery, orthopedics, urology, plastic surgery, ear-nose-throat surgery, cardiovascular surgery, ophthalmology, and anesthesiology. All study participants were asked to complete a questionnaire comprising 23 questions regarding their age, duration of work experience, appointment status, venue, and time spent on internet resources and preferred online resources for CPD purposes. In addition, participants were divided into two groups according to their appointment status: academic faculty and staff surgeons. Data analysis was performed using IBM SPSS Statistics version 17.0. The target population consisted of 216 specialists. The survey was completed by 204 (94.4%) surgical specialists. The majority of the specialists (*n* = 137, 67.2%) reported using the internet for work-related purposes every day. Daily time spent on internet resources was reported to be 30–60 min by 39.2% (*n* = 80) participants, whereas 52 (25.5%) reported spending less than 30 min. The participants wished to spend more time on internet resources. The majority of surgeons found the hospital and home equally effective in using the internet and preferred to engage alone. The mean age, English language level, usage of online resources, and the attitude score toward the perceived credibility and usefulness of e-resources were significantly higher in the academic faculty group than staff surgeons (*p* < 0.005). On the other hand, the use of Google/Google scholar was similar between the two groups (*p* = 0.192). Technical difficulties such as slow internet, need for website registration, and article fees were considered drawbacks for internet resources among all the participants.

**Conclusions:**

This study showed that most surgeons use internet resources daily for CPD and stated they would like to engage longer despite technical difficulties. Institutions should address these technical difficulties.

## Introduction

During the coronavirus disease 19 (COVID-19) epidemic, surgical training and continuous professional development (CPD) activities had to evolve to distance education, taking advantage of internet resources (1). CPD is imperative for surgeons to continuously refresh, update and improve their knowledge and skills to perform the best practices. In addition, CPD enables healthcare professionals to keep abreast with advancements in the medical and surgical fields (2). The widespread availability and powerful capabilities of the internet helped increase the incorporation of CPD into the daily routine of surgeons. With COVID-19 and social restrictions, a new era of surgical education began, consisting of online educational meetings, the usage of internet resources for mentoring, skills transfer, and even practical training. However, considering the predictable shortcomings such as lack of physical and personal interactions, the proficiency of surgical training and CPD in this era is yet to be determined (3). Other challenges of surgical education and CPD have been attributed to time constraints, patient safety concerns, and high costs.

Along with other evolving technologies such as artificial intelligence and virtual reality, internet resources hold significant promise for addressing the current challenges in surgical CPD. Furthermore, internet resources remove geographical boundaries, allowing for global sharing of knowledge, research collaboration, and tele-mentoring (4). Additionally, internet resources comprise a range of online platforms that provide anatomical illustrations, case-based learning, clinical examination, procedural skills, comprehensive course curricula, even allowing real-time peer-to-peer interactions. Thus, successful implementation of these internet-based educational tools into CPD can help surgeons keep up to date and improve their overall work-related satisfaction.

This study aimed to analyze how surgeons in a Turkish tertiary care hospital utilize online resources and their perception of these resources. In addition, technical obstacles in using internet sources were also explored. The results may help improve the proficiency of these resources and thus shape the future of surgical CPD for better educational outcomes.

## Materials and Methods

A cross-sectional study was carried out with participants from nine surgical specialties (i.e., general surgery, neurosurgery, orthopedics, urology, plastic surgery, ear-nose-throat surgery, cardiovascular surgery, ophthalmology, and anesthesiology) in a tertiary level research and training hospital in Turkey. The participants were asked to complete a questionnaire comprising 23 questions regarding their age, duration of work experience, appointment status, venue, and time spent on internet resources and preferred online resources for CPD purposes. All responses were kept anonymous. The questionnaire used was modified from MacWalter et al.’ s study (5). It comprised 23 questions in “tick all that apply”, five-point Likert style, and open-ended formats. The Turkish Surgical Association expert committee reviewed the questionnaire regarding feasibility and clarity.

The surgical staff working at the hospital has two different appointment schemes. One group is appointed as academic teaching faculty affiliated with Health Sciences University, while the other group is employed as attending surgical staff with no academic responsibilities. These two groups were compared to find whether differences existed between the faculty and staff surgeons’ preference and use of internet resources.

Data analysis was performed using IBM SPSS Statistics version 17.0 software (IBM Corporation, Armonk, NY, USA). The distributions of continuous variables were determined by the Kolmogorov-Smirnov test. The assumption of homogeneity of variances was examined by using the Levene test. As appropriate, descriptive statistics for continuous variables were expressed as means ± standard deviations (SD) or medians ± interquartile ranges (25th–75th). The number of cases and percentages were used for categorical data. The subtitle scores obtained from the questions evaluating perceived credibility and usefulness of online resources for CPD were transformed to a scale of 0 to 100 using the formula: (Subtitle Score- Lowest score) × (Range of raw score)-1 × 100.

The mean differences in ages between the groups were compared using Student’s t test. In cases where parametric assumptions were not met, the ordinal data and the continuous variables were analyzed by Mann Whitney U test. Categorical variables were analyzed using Pearson’s *χ*^2^ or Continuity corrected *χ*^2^ tests. Wilcoxon Sign Rank test was performed for intra-group comparisons. A *p* value less than 0.05 was considered statistically significant.

## Results

### Response Rates and Respondent Characteristics

The target population consisted of 216 specialists. The survey was completed by 204 surgical specialists, which comprised 94.4% of the targeted population. The mean age of all respondents was 38.8 ± 10.4 years. Most (*n* = 116; 56.9%) of the participants were younger than 40 years and male (*n* = 159, 77.9%). Duration of work experience ranged between 1 and 42 years. The participants consisted of 108 (52.9%) staff and 96 (47.1%) academic surgeons. The five most populous specialties were anesthesiology, general surgery, ear-nose-throat (ENT) surgery, orthopedic surgery, and urology (Table 1). English proficiency as a second language was self-rated by participants, and most surgeons appraised themselves as having an intermediate command of language (*n* = 81, 39.7%) (Table 1).

**Table 1:** Demographic characteristics of participants.

	*n* = 204
Age (years)	38.8 ± 10.4
Age Range	25–65
Age groups	
<40 years	116 (56.9%)
≥40 years	88 (43.1%)
Gender	
Female	45 (22.1%)
Male	159 (77.9%)
Professional experience (years)	13 (6–20)
Professional experience range (years)	1–42
Surgical Staff	
Attending surgeon	108 (52.9%)
Academic faculty	96 (47.1%)
Surgical Specialty	
General Surgery	35 (17.2%)
Neurosurgery	16 (7.8%)
Orthopedic Surgery	23 (11.3%)
Urology	23 (11.3%)
Ophthalmic Surgery	22 (10.8%)
Plastic and Reconstructive Surgery	12 (5.9%)
ENT	23 (11.3%)
Cardiovascular Surgery	15 (7.3%)
Anesthesia	35 (17.2%)
English language proficiency	
Basic	23 (11.3%)
Intermediate	81 (39.7%)
Advanced	69 (33.8%)
Superior	31 (15.2%)

### Internet usage and reasons for use

The majority of the specialists reported using the internet for work-related purposes every day (*n* = 137, 67.2%) (Table 2). Only 6 described themselves as hardly ever using the internet resources (*n* = 6, 2.9%). The three most common reasons for using the internet resources were literature review (*n* = 164, 80.4%), finding the answer to a clinical question (*n* = 156, 76.5%), and attending CPD activities (*n* = 115, 56.4%). The top preference among the internet resources was PubMed (*n* = 148, 72.5%) (Table 2). PubMed was followed by Google/Google scholar (*n* = 114, 70.6%). The other frequently used internet resources were official surgical websites (*n* = 132, 64.7%), YouTube (*n* = 107, 52.5%), Up-to-Date (*n* = 101, 49.5%) and online journals websites (*n* = 97, 47.5%). Facebook and Twitter were less frequently used (*n* = 28, 13.7%, and *n* = 16, 7.8% respectively) than the other internet resources for CPD purposes.

**Table 2:** Internet resource use and attitude reported by participants and preferred sites for continuous professional development.

Frequency of Accessing E-Resources	*n* = 204
Everyday	137 (67.2%)
Two-three times a week	38 (18.6%)
Once in a week	14 (6.9%)
Less than once in a week	9 (4.4%)
Hardly ever	6 (2.9%)
Purposes of E-Resource Use	
To inform a patient	111 (54.4%)
To answer a clinical question	156 (76.5%)
To answer a non-clinical question	107 (52.5%)
To review literature	164 (80.4%)
Continuous professional development	115 (56.4%)
Other	22 (10.8%)
Preferred E-Resources	
Official surgical websites	132 (64.7%)
Google/Google scholar	144 (70.6%)
Online journals	97 (47.5%)
PubMed	148 (72.5%)
Up-to-date	101 (49.5%)
YouTube	107 (52.5%)
Facebook	28 (13.7%)
Twitter	16 (7.8%)
Other resources	32 (15.7%)
Access Venues	
In hospital	55 (27%)
At home	43 (21.1%)
Hospital and home equally	106 (52%)
Access Setting	
Always alone	70 (34.3%)
Usually alone	117 (57.4%)
Usually with somebody	16 (7.8%)
Always with somebody	1 (0.5%)
Attitude Scores Toward the Perceived Credibility and Usefulness of E-Resources	76.7 (68.3–91.7)

Among all, 106 (52%) of the surgical specialists reported accessing internet resources at work and home equally. Fifty-five specialists (27%) reported accessing the internet mostly at hospital grounds, whereas 43 (21.1%) stated accessing primarily at home. The majority (*n* = 117, 57.4%) of the specialists reported that they preferred to be alone during internet use for CPD. The mean attitude score toward the perceived credibility and usefulness of internet resources was calculated as 76.7 [68.3–91.7].

**Figure 1:**
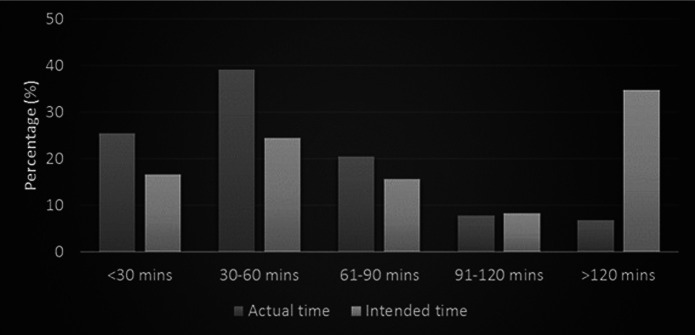
The distribution of the actual and intended time periods to use continuous professional development resources on the internet.

Daily time spent on internet resources was reported to be 30–60 min by 39.2% (*n* = 80) of the participants, whereas 52 (25.5%) reported spending less than 30 min. Also, the time spent on internet resources was significantly lower than the time the participants intended to spend (*p* < 0.001). In other words, it was observed that the participants wished to spend more time on internet resources (Figure 1).

**Figure 2:**
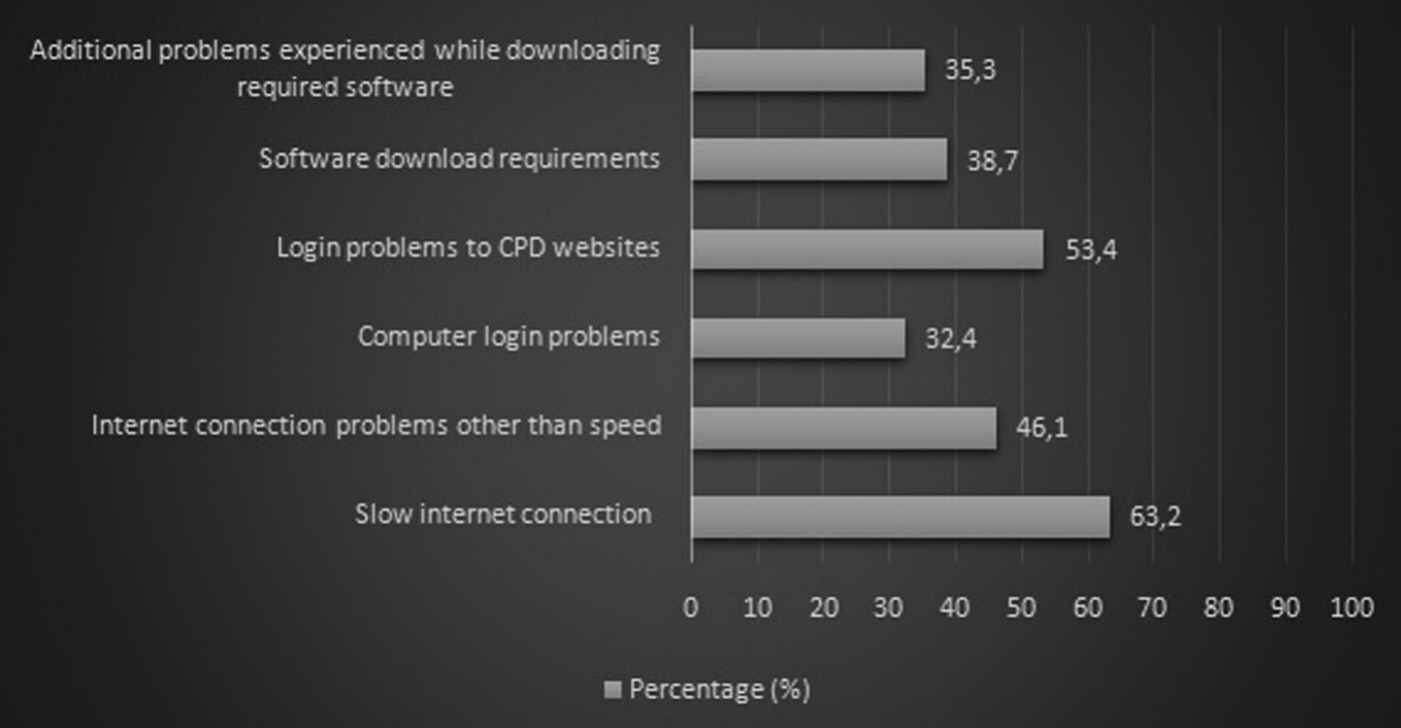
The perceived obstacles to internet resource use.

The perceived obstacles to internet resources’ use are shown in Figure 2. Our analysis elucidated that 129 (63.2%) participants listed a slow internet connection as the leading problem. The second most frequent obstacle was logging in to CPD websites (*n* = 109; 53.4%). The requirement of downloading a specific software to access CPD content was reported as an obstacle by 79 (38.7%) participants. Additional problems experienced while downloading required software were expressed by 72 (35.3%) subjects. Nearly one-third of surgeons (*n* = 66; 32.4%) reported encountering computer login problems (Figure 2).

### Intergroup Comparisons

Participants were divided into two groups according to their appointment status: academic faculty and staff surgeons. There were 96 academic faculties and 108 staff surgeons in the study group. The mean age and duration of professional experience both were significantly higher in the former group than the latter (*p* < 0.001) (Table 2). Sixty-two (64.6%) of the academic faculty classified themselves as having advanced or superior command of the English language (Table 3). However, 64.8% (*n* = 70) of the staff surgeons defined their command of the English language as basic or intermediate. This difference was statistically significant between the two groups (*p* < 0.001).

**Table 3:** Demographic characteristics of the participants according to their appointment status.

	Attending Surgeon (*n* = 108)	Academic Faculty (*n* = 96)	*p* value
Age (years)	32.4 ± 6.1	46.1 ± 9.3	**<0** **.** **001**
Age groups			**<0** **.** **001**
<40 years	92 (85.2%)	24 (25.0%)	
≥40 years	16 (14.8%)	72 (75.0%)	
Gender			0.913
Female	23 (21.3%)	22 (22.9%)	
Male	85 (78.7%)	74 (77.1%)	
Professional experience (years)	6 (4–11)	19.5 (14–30)	**<0** **.** **001**
English language skills			**<0** **.** **001**
Basic-Intermediate	70 (64.8%)	34 (35.4%)	
Advanced-Superior	38 (35.2%)	62 (64.6%)	

*Bold p values indicate significant difference.*

Comparative analysis elucidated that the academic participants used online resources more frequently (*p* < 0.001). They were significantly more frequently used to inform a patient, review the literature, and for CPD purposes (*p* = 0.014, ***p*** < 0.001, ***p*** < 0.001, respectively). Academic faculty reported using official surgical websites, online journals, PubMed, Up-To-Date, YouTube, Facebook, and Twitter significantly more frequently than the staff surgeons (Table 4). Google/Google scholar use was similar between these two groups (*p* = 0.192). The attitude score toward the perceived credibility and usefulness of e-resources was significantly higher in the academic faculty group (*p* < 0.001).

**Table 4:** Comparison of academic and non-academic participants’ attitude and use of online continuous professional development resources.

	Attending Surgeon (*n* = 108)	Academic Faculty (*n* = 96)	*p* value
Frequency of Accessing E-Resources			**<0** **.** **001**
Everyday	62 (57.4%)	75 (78.1%)	
Two-three times a week	21 (19.4%)	17 (17.7%)	
Once a week	14 (13.0%)	0 (0.0%)	
Less than once a week	7 (6.5%)	2 (2.1%)	
Hardly ever	4 (3.7%)	2 (2.1%)	
Purposes of E-Resource Use			
To inform a patient	50 (46.3%)	61 (63.5%)	**0** **.** **014**
To answer a clinical question	81 (75.0%)	75 (78.1%)	0.719
To answer a non-clinical question	51 (47.2%)	56 (58.3%)	0.113
To review literature	76 (70.4%)	88 (91.7%)	**<0** **.** **001**
* Continuous Professional development*	48 (44.4%)	67 (69.8%)	**<0** **.** **001**
* Other*	4 (3.7%)	18 (18.8%)	**<0** **.** **001**
Preferred E-Resources			
Official surgical websites	57 (52.8%)	75 (78.1%)	**<0** **.** **001**
Google/ Google scholar	72 (66.7%)	72 (75.0%)	0.192
Online Journals	34 (31.5%)	63 (65.6%)	**<0** **.** **001**
PubMed	63 (58.3%)	85 (88.5%)	**<0** **.** **001**
Up-To-Date	41 (38.0%)	60 (62.5%)	**<0** **.** **001**
Youtube	46 (42.6%)	61 (63.5%)	**0** **.** **003**
Facebook	7 (6.5%)	21 (21.9%)	**0** **.** **003**
Twitter	2 (1.9%)	14 (14.6%)	**0** **.** **002**
Other resources	13 (12.0%)	19 (19.8%)	0.184
Attitude Scores Toward the Perceived Credibility and Usefulness of E-Resources	73.3 (62.1–80.8)	85.8 (75.0–97.9)	**<0** **.** **001**

*Bold p values indicate significant difference.*

## Discussion

Successful implementation of internet-based educational tools into CPD requires formal needs assessment, collaborative efforts of educational and professional bodies, and rigorous evaluation of their effectiveness. *Internet* is a widely used technology that may provide a novel learning modality for surgeons (6, 7). During the COVID-19 pandemic, the traditional surgical education and CPD activities were suspended. These were replaced by online discussions, interactions, and training, allowing continuous distribution of knowledge and experience (1, 3, 8). Moreover, social media offers a range of interactive online platforms with users worldwide (9).

Most of the (*n* = 116; 56.9%) surgeons in our study were younger than 40, and their work experience was less than 15 years, representing the new generation of surgeons. They reported internet use for professional development at least 30–60 min daily and desired to spend more time provided they had more time and were free of technical problems.

The most preferred internet resources were PubMed and Google/Google Scholar. The academic faculty responsible for residency and fellowship training used PubMed more frequently than the staff surgeons. This difference may be due to the limited English proficiency in the latter group, as delineated in the questionnaire results. However, this difference was not present with Google/Google Scholar use. Since Google creates content in multiple languages, it may be preferred more by surgeons with limited English proficiency.

The most frequent technical problem reported by the surgeons was the slow internet connection. Improving internet connection and speed institutionally can solve this problem. Another option would be to create wireless hotspots within the hospital grounds where surgeons can access online resources easier and faster. For more comprehensive access and consumption of internet resources, professional development sites should consider reducing the membership and registration fees (10). PubMed and Google/ Google Scholar were able to eliminate these fees and thus are the two most preferred online resources by surgeons (11). A solution to circumvent the membership cost is to provide institutional registrations for the surgeons (12). In order to obviate the additional software download requirement, which was encountered by 38.7% of participants, the CPD websites should utilize frequently used interfaces while designing online educational content.

Perceived obstacles to internet resource access are in close relationship with digital competence (13, 14). *Digital competence* is a relatively new term used to explain a person’s ability to perform digital tasks, read digital data, and apply new knowledge obtained from digital environments (15). Digital competence is a fundamental skill for surgeons’ CPD activities. Thus, mentorship or basic skills review may improve the surgeons’ attitude towards the credibility of internet resources. Van der Vaart et al. stated that good digital competence was based on academic skills such as reading and writing (14). This finding might explain the academic faculty’s more frequent use of internet resources.

Our data showed that 67.2% of the surgeons used the internet resources daily despite all challenges. However, this rate is lower than the frequency of internet resource consumption reported by the general practitioners of Denmark and Scotland (5).

On the other hand, the reasons for internet resource use were parallel to those reported in our study: Mainly to review the literature and answer a clinical question. This finding suggests that internet resource use and demand among different health care systems are similar. These similarities may assist in generalizing the results of our study beyond the surgical community.

Facebook and Twitter were not commonly preferred by surgeons for CPD (13.7% and 7.8%, respectively). However, these platforms may help professionals collaborate with each other, participate in journal clubs and join online discussions. A recent study that included oncologists revealed that social media was especially preferred for networking, research sharing, and leadership development (16). On the other hand, YouTube was frequently used by the surgeons participating in our study. This finding is probably due to the demonstrative surgical videos where one can watch and learn the technical details and pitfalls of a surgical procedure (17, 18). Farag et al. emphasized the increased use of YouTube among surgical trainees, recommending expert surgeons to register to YouTube and share their videos and make comments on others (17).

There are several limitations to this study. First, its results cannot be generalized to the surgical specialists since the survey was undertaken at a single university-affiliated tertiary care center in Ankara, the capital of Turkey. Second, the study was conducted at the beginning of the COVID-19 pandemic and provided cross-sectional data; however, with the extension of the pandemic and restrictions, the use of internet resources might have increased since the time this study was conducted.

This study showed that most surgeons use the internet daily for CPD and wish to engage longer despite technical difficulties. This study determined that most surgeons found the hospital and home equally effective in using internet resources and preferred to engage with the content alone. They reported preferring primarily PubMed, Google/Google Scholar, and official surgical websites as their CPD resource and stated that they would like to engage with the content longer despite the technical difficulties. To improve the efficacy of internet resource use for surgeons, the technical problems defined in this article should be tackled by institutions individually.

## Data Availability

The original contributions presented in the study are included in the article/Supplementary Material, further inquiries can be directed to the corresponding author/s.

## References

[1] Wijesooriya NR Mishra V Brand PLP Rubin BK. COVID-19 and telehealth, education, and research adaptations. Paediatr Respir Rev. (2020) 35:38–42. 10.1016/j.prrv.2020.06.009 32653468 PMC7301824

[2] Lin J Reddy RM. Teaching, mentorship, and coaching in surgical education. Thorac Surg Clin. (2019) 29:311–20. 10.1016/j.thorsurg.2019.03.008 31235300

[3] Kitto S. Continuing professional development in the era of COVID-19. J Contin Educ Health Prof. (2020) 40:73. 10.1097/CEH.0000000000000298 32472808

[4] Fuertes-Guiró F Viteri Velasco E. Ethical issues in surgical tele mentoring: challenges and dilemmas of an innovative technology. Minerva Chir. (2018) 73:347–9. 10.23736/S0026-4733.18.07566-1 29397635

[5] MacWalter G McKay J Bowie P. Utilization of internet resources for continuing professional development: a cross-sectional survey of general practitioners in Scotland. BMC Med Educ. (2016) 16:24. 10.1186/s12909-016-0540-5 26791566 PMC4721189

[6] Maertens H Madani A Landry T Vermassen F Van Herzeele I Aggarwal R. Systematic review of e-learning for surgical training. Br J Surg. (2016) 103:1428–37. 10.1002/bjs.10236 27537708

[7] Vogelsang M Rockenbauch K Wrigge H Heinke W Hempel G. Medical education for “Generation Z”: everything online?*! -* An analysis of internet-based media use by teachers in medicine. GMS J Med Educ. (2018) 35:Doc21. 10.3205/zma001168 29963611 PMC6022581

[8] Alsoufi A Alsuyihili A Msherghi A Elhadi A Atiyah H Ashini A Impact of the COVID-19 pandemic on medical education: medical students’ knowledge, attitudes, and practices regarding electronic learning. PLoS One. (2020) 15:e0242905. 10.1371/journal.pone.0242905. 33237962 PMC7688124

[9] Markham MJ Gentile D Graham DL. Social media for networking, professional development, and patient engagement. Am Soc Clin Oncol Educ Book. (2017) 37:782–7. 10.1200/EDBK_180077 28561727

[10] Dunbar GL Symonds L. Expanding collaborations between the neuroscience training committee of the society for neuroscience and the faculty for undergraduate neuroscience. J Undergrad Neurosci Educ. (2018) 16:A273–6. 30254543 PMC6153017

[11] Voronin Y Myrzahmetov A Bernstein A. Access to scientific publications: the scientist's perspective. PLoS One. (2011) 6:e27868. 10.1371/journal.pone.0027868. 22114716 PMC3219702

[12] Massarrat S Kolahdoozan S. Critical assessment of progress of medical sciences in iran and turkey: the way developing countries with limited resources should make effective contributions to the production of science. Arch Iran Med. (2011) 14:370–7. 22039839

[13] Konttila J Siira H Kyngäs H Lahtinen M Elo S Kääriäinen M Healthcare professionals’ competence in digitalization: a systematic review. J Clin Nurs. (2019) 28:745–61. 10.1111/jocn.14710 30376199

[14] van der Vaart R Drossaert C. Development of the digital health literacy instrument: measuring a broad spectrum of health 1.0 and health 2.0 skills. J Med Internet Res. (2017) 19:e27. 10.2196/jmir.6709. 28119275 PMC5358017

[15] Foadi N Varghese J. Digital competence - a key competence for todays and future physicians. J Eur CME. (2022) 11:2015200. 10.1080/21614083.2021.2015200. 34992949 PMC8725739

[16] Morgan G Tagliamento M Lambertini M Devnani B Westphalen B Dienstmann R Impact of COVID-19 on social media as perceived by the oncology community: results from a survey in collaboration with the European Society for Medical Oncology (ESMO) and the OncoAlert Network. ESMO Open. (2021) 6:100104. 10.1016/j.esmoop.2021.100104. 33838532 PMC8038939

[17] Farag M Bolton D Lawrentschuk N. Use of YouTube as a resource for surgical education-clarity or confusion. Eur Urol Focus. (2020) 6:445–9. 10.1016/j.euf.2019.09.017 31606471

[18] Al-Khatib TA. Surgical education on YouTube. Saudi Med J. (2014) 35:221–3. 24623200

